# Unraveling the Nature of Vibrational Dynamics in CsPbI_3_ by Inelastic Neutron Scattering and Molecular Dynamics Simulations

**DOI:** 10.1021/acs.jpclett.5c00778

**Published:** 2025-05-08

**Authors:** Rasmus Lavén, Erik Fransson, Paul Erhart, Fanni Juranyi, Garrett E. Granroth, Maths Karlsson

**Affiliations:** † Department of Chemistry and Chemical Engineering, Chalmers University of Technology, SE-412 96 Göteborg, Sweden; ‡ Department of Physics, Chalmers University of Technology, SE-412 96 Göteborg, Sweden; § Laboratory for Neutron Scattering and Imaging, PSI Center for Neutron and Muon Sciences, Forschungsstrasse 111, 5232 Villigen, Switzerland; ∥ Neutron Scattering Division, Neutron Sciences Directorate, Oak Ridge National Laboratory, Oak Ridge, Tennessee 37831, United States

## Abstract

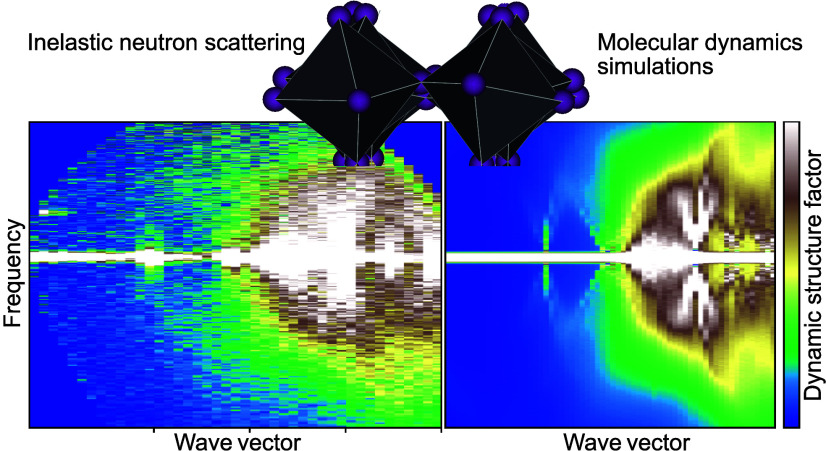

Cesium lead iodide, CsPbI_3_, is an optoelectronic material of large interest for various technological applications; however, fundamental questions surrounding the vibrational dynamics of this material, especially regarding its role in structural phase transitions, remain to be elucidated. Here, in a combined variable temperature inelastic neutron scattering (INS) and machine-learning based molecular dynamics (MD) simulation study, we show that the stable phase at room temperature, i.e., the nonperovskite δ-phase, exhibits phonon modes with weak anharmonicity with only a weak temperature dependence from 10 K all the way up to the transition to the cubic perovskite α-phase at approximately 600 K. In contrast, the α-phase features anharmonic and damped vibrational dynamics, mainly associated with overdamped tilting motions of the PbI_6_ octahedra. Crucially, these overdamped tilting modes, which relate to the tetragonal and orthorhombic distorted perovskite phases (β- and γ-phase, respectively) formed at lower temperatures, stay overdamped by more than 100 K above the respective phase transition. This suggests a flat energy landscape of octahedral tilting motions in α-CsPbI_3_ and with structural fluctuations on the picosecond time scale with tilting patterns that locally resemble the structure of the β- and γ-phases. The vibrational dynamics of α-CsPbI_3_ are also characterized by pronounced anharmonic motions with large thermal displacements of the Cs^+^ ions, but these modes remain underdamped at 600 K.

Metal halide perovskites (MHPs), i.e., materials of the form *ABX*_3_, where *A* and *B* are cations and *X* is a halide anion, are a class of materials that have gained great attention in recent years for their promise in optoelectronic applications, such as solar panels and lighting.^1,2^ On a fundamental level, one of the general characteristics of these materials is their generally “soft” nature, with typically unusually large thermal displacements of the constituting atoms. Early studies suggested that these large thermal displacements are present mainly in hybrid organic–inorganic MHPs and are related to dynamics of the organic cations, which express themselves as various reorientational motions.^3^ However, more recent works have shown that large thermal displacements seem to be generally present also in all-inorganic MHPs, such as CsPbBr_3_^4^ and have, in part, been related to soft and, typically, overdamped vibrational dynamics in these materials. Such soft, or overdamped dynamics, are commonly observed in the vicinity of displacive phase transitions of many materials, such as, e.g., SrTiO_3_,^5^ BaTiO_3_,^6^ LiNbO_3_,^7^ and CsPbCl_3_.^8^ As one lowers the temperature and moves away from the phase transition into the lower symmetry crystal phase, these soft modes “freeze in” and become static, giving rise to the elastic Bragg scattering of the supercell reflections in the lower-symmetry phase. Crucially, overdamped vibrational dynamics in MHPs have also been related to the formation of large polarons and screening of charge carriers, defect tolerance, moderate charge carrier mobility, and low radiative recombination rates, that are of high importance for MHPs in actual applications.^9,10^ This suggests that the vibrational dynamics in MHPs are a key to understanding the underpinning mechanisms of their optoelectronic properties. The current understanding of the nature of vibrational dynamics, especially overdamped dynamics and its relevance for optoelectronic properties in MHPs, comes mostly from INS, Raman spectroscopy, and computer simulations.^4,11−15^ Phase transitions and dynamics in halide perovskites have been studied prior using computational and theoretical methods.^16−20^ With regard to all-inorganic MHPs, most work has been focused on CsPbBr_3_, for which an INS study on a single crystal sample unraveled overdamped vibrational dynamics related to PbBr_6_ octahedral tilting motions at the M and R points of the Brillouin zone that, in turn, relate to the cubic-to-tetragonal and tetragonal-to-orthorhombic phase transitions, respectively.^15^ Recently, some of us showed by MD simulations that these octahedral tilting motions stay overdamped far above the phase transition temperature,^21^ which is related to the flat energy landscape present in the cubic perovskite phase of CsPb*X*_3_ (*X* = I, Br).^11,12,22−26^ However, the theoretical work^21^ requires consolidation and, in particular, motivates further research aimed at unraveling whether these results are also applicable to other MHP materials. In this work, we thus investigate the nature of vibrational dynamics in the halide-ion-substituted material CsPbI_3_ over a large temperature range from 10 to 600 K, using a combination of INS and MD simulations. CsPbI_3_ adopts an orthorhombic (δ) nonperovskite phase (space group *Pnma*) at room temperature, which transforms to a cubic (α) perovskite phase (space group *Pm*3̅*m*), i.e., the optically active phase, upon heating to around 600 K.^12^ By cooling from 600 K, the material has been shown to transform to a tetragonal (β) phase (space group *P*4/*mbm*) at 554 K and to an orthorhombic (γ) phase (space group *Pnma*) at 457 K,^12^ although it should be noted that the direct transition upon cooling from the α-phase to the δ-phase has also been observed.^27^

Up to now, the only experimental reports on the vibrational dynamics in CsPbI_3_ are based on results from Raman spectroscopy measurements; however, these are in most cases limited to the study of symmetry-allowed optical vibrational modes at the center of the Brillouin zone.^28−32^ In our work, this is mitigated by the use of INS since INS is sensitive to all vibrational modes in a material and, furthermore, can be compared to MD simulation data on an absolute scale. In effect, this means that INS data can provide a stringent test of the calculated local structures and dynamics present in the material. The combined analyses of variable temperature INS and MD simulation data thus allow us to unravel the nature of vibrational dynamics of CsPbI_3_ and how it changes with temperature. The INS experiments were carried out on a powder sample of CsPbI_3_, from which we derived the dynamical structure factor *S*(*q*, ω), where ω and *q* are, respectively, the energy transfer and modulus of the wavevector transfer; see the Methods section for details about the experiments and MD simulations.

Figure 1 compares the experimental dynamical structure factor *S*(*q*, ω) for the nonperovskite (δ) phase (at 550 K) and the cubic perovskite (α) phase of CsPbI_3_ (at 600 K). Figure S2 shows the corresponding *S*(*q*), which confirms the δ-to-α phase transition between 550 and 600 K. For both phases, *S*(*q*, ω) is characterized by strong, *q*-dependent, coherent scattering. For the nonperovskite phase, δ-CsPbI_3_, this coherent scattering is manifested as acoustic phonon scattering, which disperses from the (212) Bragg peak at around *q* = 1.85 Å^–1^^33^ up to about 3 meV, whereas essentially no phonon scattering is observed below *q* = 1.85 Å^–1^. These phonons exhibit only a weak temperature dependence from 10 K up to the phase transition to the α-phase around 600 K (see Figure S6). For the perovskite phase, α-CsPbI_3_, acoustic phonon scattering dispersing out from the Bragg peaks can be also observed, with low intensity of the phonon scattering below the (110) Bragg peak at approximately *q* = 1.4 Å^–1^. However, a striking difference between the two phases is the marked increase in the level of low-energy, virtually quasielastic scattering for α-CsPbI_3_. This is further evident in Figure 1c, which compares the *q*-integrated *S*(*q*, ω) for both phases of CsPbI_3_. Included in this graph is the simulated *S*(*q*, ω), as obtained from MD simulations for both phases and at the same temperatures. We observe excellent agreement between the experimental and simulated data, providing strong support that the simulated *S*(*q*, ω) can be used to gain detailed insight into the underlying dynamics.

**Figure 1:**
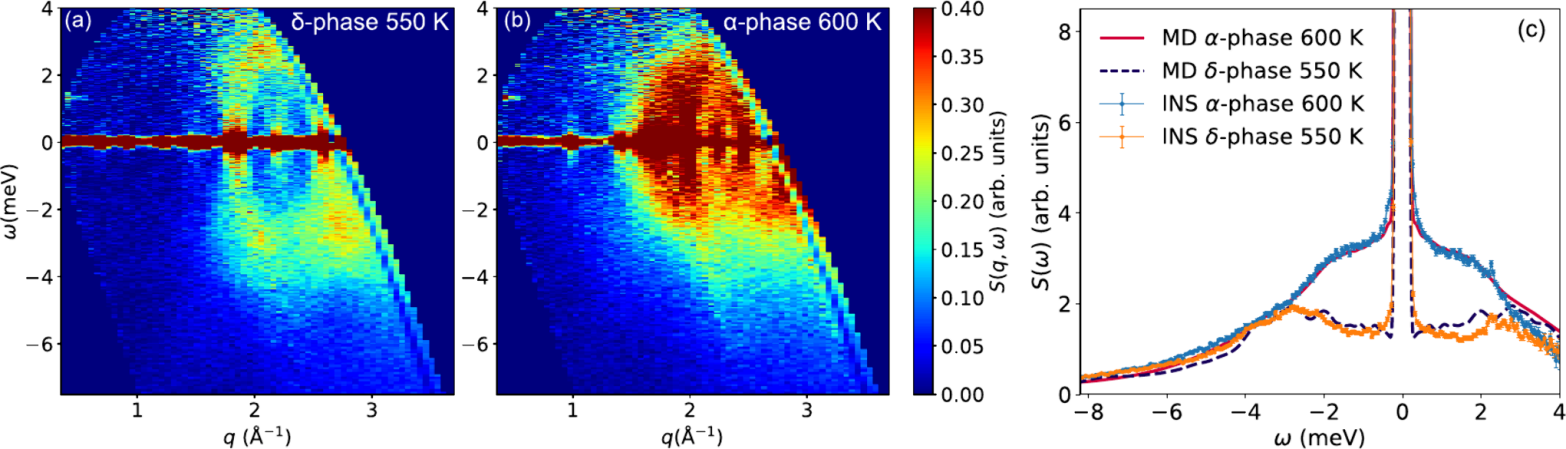
*S*(*q*, ω) of δ-CsPbI_3_ (a) and α-CsPbI_3_ (b), as measured on FOCUS. The small peak at 1.33 meV for very small *q* (≲0.5 Å^–1^) is a spurious peak from the instrument. (c) Comparison of the experimental and simulated *S*(ω), here summed over all measured *q* values (∼0.2 to 3.5 Å^–1^). The MD simulated data is convoluted with a Gaussian of fwhm = 0.15 meV to mimic the contribution of the instrumental resolution in the experimental data.

Since it is very unlikely that α-CsPbI_3_ possesses any translational and/or rotational diffusional dynamics and due to the strong *q*-dependence, the quasielastic scattering signal suggests the presence of relaxational dynamics associated with either strongly damped and/or overdamped phonon dynamics. To investigate the origin of the dynamics giving rise to quasi-elastic scattering, we compare the experimental *S*(*q*, ω) to the computed partial dynamical structure factors for the Cs–Cs, Pb–Pb, and I–I correlations for both δ-CsPbI_3_ (at 550 K) and α-CsPbI_3_ (at 600 K; Figure 2). For α-CsPbI_3_, the partial dynamical structure factors are manifested by quasi-elastic scattering due to I–I correlations, whereas dynamics associated with Cs–Cs and Pb–Pb correlations are generally characterized by higher energies and are less pronounced. Notably, the quasielastic scattering due to I–I correlations is concentrated to the *q*-ranges of about 1.5 Å^–1^ to 1.9 Å^–1^ and 2.85 Å^–1^ to 3.2 Å^–1^, which suggests that the dynamics are associated with overdamped octahedral tilting modes at the M and R points of the Brillouin zone. This is supported by the calculated current correlations along the high-symmetry directions (Figure S17), which shows overdamped modes along the M–R lines in the Brillouin zone. This is in full accordance with earlier studies of CsPbBr_3_.^15,21^ Note, the cross-correlation terms (Cs–I, Pb–I, and Cs–Pb) are generally slightly smaller than the noncross terms over the whole probed wavevector range, although it should be noted that also the Cs–I correlations contribute to the quasielastic scattering at 1.5 Å^–1^ to 1.9 Å^–1^ and 2.85 Å^–1^ to 3.2 Å^–1^ (Figures S14 and S15).

**Figure 2:**
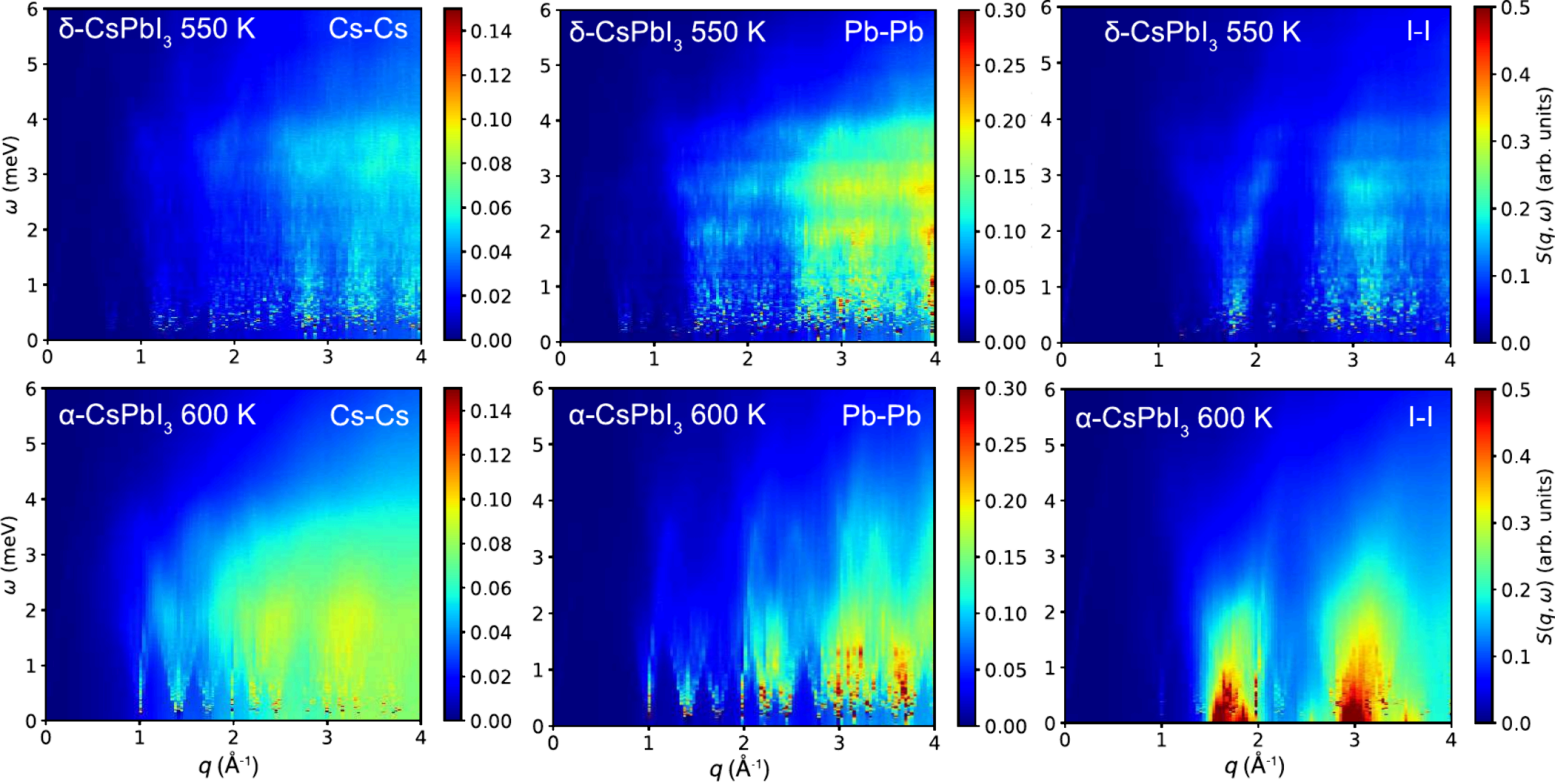
Partial dynamic structure factors of δ-CsPbI_3_ at 550 K (top panels) and α-CsPbI_3_ at 600 K (bottom panels) as derived from MD simulations. Note that the different elements are plotted on different scales for *S*(*q*, ω). The elastic data point at ω = 0 is removed from the data.

Figure 3a–c shows fits to the *S*(*q*, ω) spectra of α-CsPbI_3_ at *q* = 1.55 Å^–1^, 1.65 Å^–1^, and 1.85 Å^–1^ (the *q*-integration range is ±0.05 Å^–1^). The two first *q*-values correspond to the values close to the modulus of , i.e., |**M**| = 1.58 Å^–1^ and , i.e., |**R**| = 1.66 Å^–1^. The third *q*-value corresponds to a value close to the modulus of both , i.e., |**X**| = 1.8 Å^–1^, and , i.e., |**M**| = 1.87 Å^–1^. It implies that the data in Figure 3a–c contain a large contribution from phonons at these high-symmetry points, although it must be noted that also phonons at all other reciprocal lattice vectors of the same modulus will contribute to the spectra. An illustration of the parts of *q*-space probed with the powder INS at *q* = 1.55 Å^–1^ and *q* = 1.85 Å^–1^ is shown in Figure S12.

**Figure 3:**
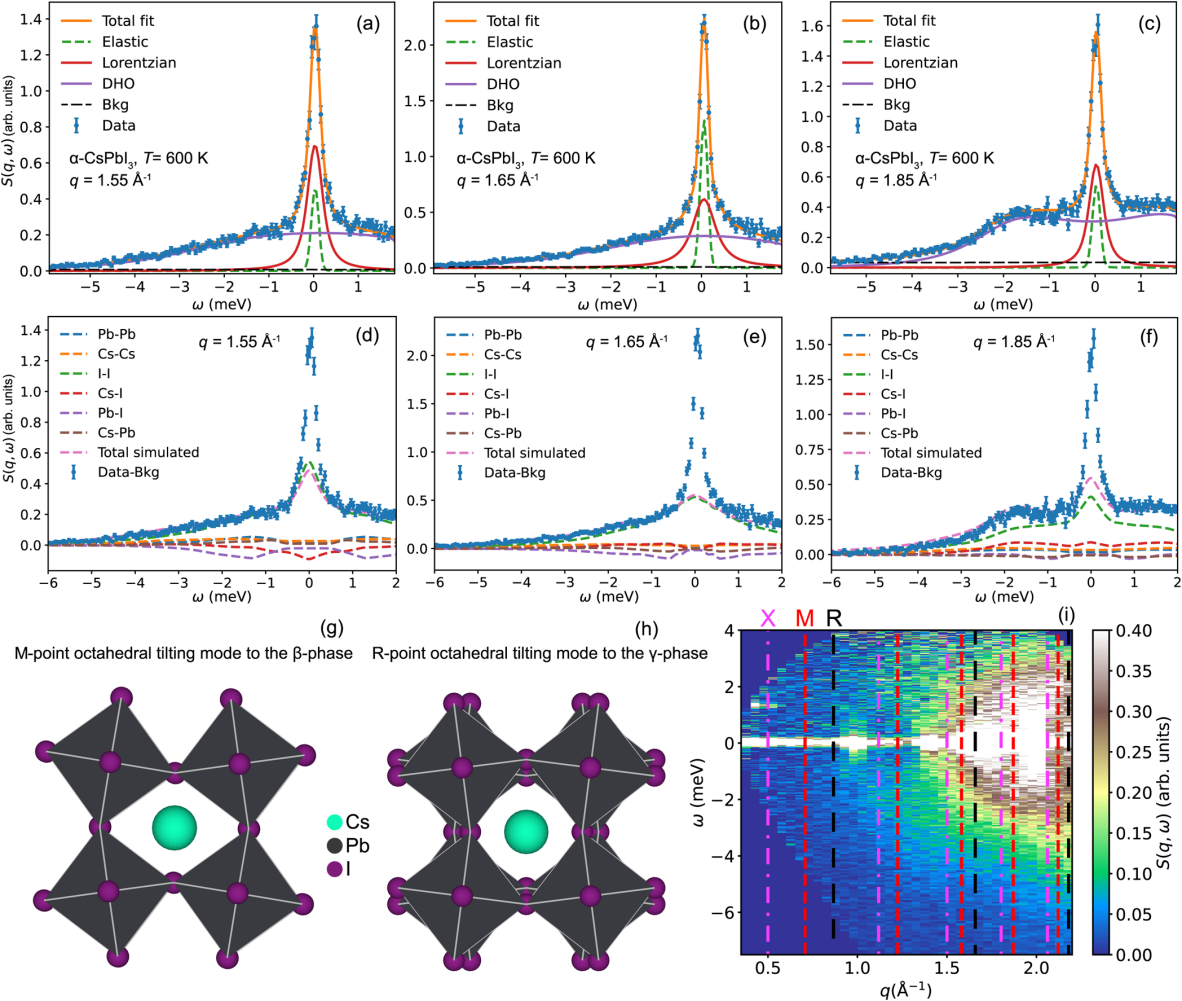
(a–c) *S*(*q*, ω) of α-CsPbI_3_ at 600 K together with fits for *q* = 1.55 Å^–1^ and 1.65 Å^–1^ and 1.85 Å^–1^. The data was measured on FOCUS. (d–f) Same data as in (a–c) but compared to the partial *S*(*q*, ω) from the MD simulations. The MD simulated data is convoluted with a Gaussian of fwhm = 0.15 meV to mimic the contribution of the instrumental resolution in the experimental data. The higher elastic scattering in the experimental data is due to incoherent elastic scattering which is not considered in the simulation (see Figure S11). (g–h) Illustration of the octahedral tilting mode at the M- and R-points. Note that, for the M-point mode, the layers tilt in the same direction, while for the R-point mode, the alternating layers tilt in alternating directions. The figures are made using VESTA.^35^ (i) *S*(*q*, ω) of α-CsPbI_3_ at 600 K with the modulus of different high-symmetry points of the cubic Brillouin zone (X, M, R) marked as dashed lines.

At *q* = 1.55 Å^–1^ (Figure 3a), i.e., at the M-point of the Brillouin zone, the quasielastic contribution to *S*(*q*, ω) can be adequately fitted to one Lorentzian function with a spectral line width of 0.32 meV at full width at half-maximum (fwhm). The remaining inelastic scattering can be approximated by a damped harmonic oscillator (DHO) function. However, since we measured on a powder sample and, therefore, measure the average of *S*(*q*, ω) over all wavevector directions, the DHO component will correspond to an average of many phonons lying close in energy and not related to a single real dynamical process; therefore, we do not analyze the DHO component further in this work. The Lorentzian component is assigned to octahedral tilting motions, with a relaxational time of 2*ℏ*/fwhm ≃ 4 ps, which, because of the quasielastic nature of the scattering, are overdamped in nature, in perfect agreement with the MD simulations; see Figure 3d–f for a comparison between MD simulated partial *S*(*q*, ω). The higher elastic scattering in the experimental data is due to incoherent elastic scattering, which is not considered in the simulation (see Figure S11).

Notably, these tilting modes are related to the α-to-β phase transition, which is expected to occur at ∼550 K for CsPbI_3_,^12^ so quasielastic scattering at the M-point is indeed expected near this temperature based on the soft-phonon model for displacive phase transitions.^34^ Remarkably, however, we observe such quasielastic scattering about 50 K above the phase transition temperature, which consolidates the earlier MD simulation study on CsPbBr_3_ in that the octahedral tilting motions are overdamped even far above (more than 100 K) the phase transitions.^21^ A graphical illustration of the M-point mode is shown in Figure 3g.

Similarly to the data at *q* = 1.55 Å^–1^, at *q* = 1.65 Å^–1^ [Figure 3b], i.e., at the R-point of the Brillouin zone, the quasielastic scattering of *S*(*q*, ω) can be fitted to one Lorentzian component, but here, the Lorentzian function corresponds to slightly faster-time scale dynamics (2.4 ps). The tilting mode at R corresponds to the tilt observed at the phase transition to the orthorhombic (γ) phase, which is expected to occur around 450 K^12^ (Figure 3h). In the soft phonon model, the line width would increase as one moves away from the critical temperature; thus, it is reasonable that the relaxational dynamics related to the quasielastic scattering is faster around R. Nevertheless, it is remarkable to observe the quasielastic scattering at 600 K, which is 150 K above the transition to the orthorhombic (γ) phase.

At *q* = 1.85 Å^–1^ (Figure 3c), the overdamped dynamics feature a relaxation time of 4.7 ps, which is similar to the overdamped dynamics associated with octahedral tilting motions at *q* = 1.55 Å^–1^. In this context, we note that X point phonons are not expected to be soft close to the phase transitions; hence, they cannot be related to such a mode.^15^ Instead, the relaxational dynamics observed here are most likely to come from scattering at the next M-point at , which is featured by a modulus of 1.87 Å^–1^. As inferred from the partial *S*(*q*, ω) as calculated from the MD simulations [Figures 2 and 3f], in addition to the I–I correlations, the Cs–Cs and Cs–I correlations contribute more at this higher *q*-value. Consequently, these underdamped dynamics also contain a contribution from displacement of the Cs^+^ ions. Detailed information about these dynamics can be obtained by comparing the partial dynamic structure factors. More specifically, the partial *S*(*q*, ω) for Cs–Cs correlations exhibits a sharp increase in intensity at around ≈1.6 Å^–1^, above which it is roughly *q*-independent (incoherent). This gives evidence of low-energy (soft) spatially decoherent motions of the Cs^+^ cations in the perovskite cage at 600 K.

With regard to the difference in vibrational dynamics between δ and α-CsPbI_3_, we note that the average Pb–I bond lengths are almost the same in both phases (3.2 Å and 3.14 Å for δ- and α-phase, respectively) and the average Cs–I bond length on the other hand differs significantly (3.9 Å and 4.45 Å for the δ- and α-phase, respectively).^12,33^ At the same time, the shortest average Pb–Cs distance is larger in the perovskite phase (4.92 Å and 5.45 Å for the δ- and α-phase, respectively). This could indicate a significantly different potential energy landscape for the Cs^+^ ions in the perovskite phase, with a lower interaction potential and larger atomic displacements, which is in agreement with computational studies showing a flat energy landscape in perovskite CsPbI_3_.^11,12^ It also explains the large thermal displacement parameters of Cs (isotropic) and I (perpendicular to the Pb–I–Pb bonds) in the cubic perovskite phase, which was found to be significantly smaller in the orthorhombic δ-phase.^27^ These large thermal displacements of I and Cs atoms would also mean that the distribution of bond lengths is much broader for Cs and I atoms in the cubic α-phase. A similar conclusion has been reached for isostructural CsSnI_3_ for which it was found that the phase transition to the cubic perovskite phase is derived from the stabilization of “rattling” Cs^+^ cations within the cubo-octahedral sites.^36^ Interestingly, a recent study based on the analysis of X-ray pair distribution functions suggests that the rattling of Cs^+^ ions, low number of Cs–I contacts, and high degree of octahedral distortion cause the instability of the perovskite phase CsPbI_3_.^37^ However, another study has shown that there is a well-defined phase relation between the Cs^+^ dynamics and the PbI_6_ framework dynamics in the orthorhombic γ-phase.^38^ Here, we directly observe such behavior of Cs^+^ cations in α-CsPbI_3_. It is clear from the *q*-dependence of the partial Cs–Cs *S*(*q*, ω) from the MD data [cf. Figure 2 and Figure S9] that the dynamics of the Cs^+^ cations become significantly more spatially incoherent in the α-phase compared to the δ-phase and also compared to the other atomic species in the material. However, one should note that the calculated *S*(*q*, ω) shows that the dynamics of Cs^+^ cations, although heavily damped, remain underdamped in α-CsPbI_3_.

The observation of quasielastic scattering related to overdamped dynamics of octahedral tilting motions in α-CsPbI_3_ is in full accordance with our results for CsPbBr_3_ (Figure S13), suggesting no strong dependence of the type of halide ion (*X*) on the overall vibrational dynamics in CsPb*X*_3_ (*X* = I and Br). Furthermore, we note that the extracted relaxation time of the overdamped dynamics is about 4 ps, which means that the cubic α-phase of CsPbI_3_ exhibits structural fluctuations with tilting patterns that locally resemble the tetragonal and orthorhombic distorted structures on the ps time scale.

To conclude, our INS and MD simulation studies of CsPbI_3_ are in excellent agreement and have unraveled the nature of vibrational dynamics in the various phases of CsPbI_3_. Crucially, we have shown that the stable phase at room temperature, i.e., in the nonperovskite δ-phase, exhibits vibrational dynamics with only a weak temperature dependence from 10 K all the way up to the transition to the cubic perovskite α-phase at approximately 600 K. In contrast, the α-phase features anharmonic and damped vibrational dynamics, mainly associated with overdamped tilting modes of PbI_6_ octahedra, with relaxational times between 2 and 4 ps at 600 K. The overdamped dynamics highlight the flat energy landscape for octahedral tilting motions in α-CsPbI_3_, even markedly above the phase transition temperature from lower symmetry distorted orthorhombic and tetragonal perovskite phases (γ- and β-phases, respectively), which thus give rise to dynamical fluctuations on the picosecond time scale in the α-phase. These dynamical fluctuations may explain the discrepancy found in the literature between the average cubic structure found by diffraction and the local, noncubic structure, as observed with total-scattering and spectroscopy techniques. Our results thus show that information about the vibrational dynamics can even provide crucial information for understanding the structure of these types of materials.

Finally, one should note that INS was performed on a powder sample, whereas the other INS studies on MHPs of this kind were performed on single crystal samples. While, in principle, the INS signal of a powder sample is less informative, since the signal is averaged over all *q* directions, the combination with MD simulations allows us to still draw detailed conclusions about the dynamics in the material. In effect, our results highlight that this approach can be applied to studies of other MHPs, as well as other solid-state materials, which may not be possible to synthesize as (large enough) single crystals. This is in particular true for the here studied material CsPbI_3_, for which the presence of the thermodynamically stable nonperovskite δ-phase at room temperature makes the synthesis of single crystals of perovskite phase CsPbI_3_ more challenging.^33^

## Methods

### Experimental Details

The INS experiments were carried out on the time-of-flight spectrometers FOCUS^39^ at the Swiss Spallation Neutron Source SINQ and ARCS^40^ at the Spallation Neutron Source in the U.S. The samples, CsPbI_3_ and CsPbBr_3_ powders, were held inside standard Al cylindrical shaped samples cells, which were kept unsealed to keep the samples under dynamic vacuum during the measurements; see Supporting Information (SI) for further details about the samples.

FOCUS was set up using an incident neutron wavelength of 4 Å (corresponding to an energy of 5.11 meV), which yielded an energy resolution at fwhm of approximately 0.2 meV and a wavevector transfer (*q*) range of 0.5 Å^–1^ to 2.7 Å^–1^ at the elastic line. Standard data reduction of the measured raw data was performed within the DAVE software^41^ and included normalization to a vanadium standard, for detector efficiency correction, and subtraction of the scattering from an empty sample cell. ARCS was set up using incident neutron energies of 15, 30, and 50 meV, which provided corresponding energy resolutions at fwhm of 0.45, 0.9, and 1.5 meV at the elastic line. Standard data reduction of the measured raw data was performed within the Mantid software^42^ and included the same corrections as for the FOCUS data. For both experiments, the computed response function is the dynamic structure factor *S*(*q*, ω). The energy and momentum transfer *E* = ℏω and |**p**| = ℏ*q* will be used interchangeably with ω and *q*.

The quasielastic scattering from overdamped dynamics in *S*(*q*, ω) was fitted to a Lorentzian centered around ω = 0. In addition to the Lorentzian component, an elastic scattering component, described by a delta function convoluted with the instrumental resolution function (as here determined by a measurement of a vanadium standard), a DHO function used to describe the remaining inelastic scattering, and a constant background (Bkg) were included in the fitting. The Lorentzian and DHO components were also convoluted with the instrumental resolution function in the fitting.

### Computational Details

The experimental *S*(*q*, ω) was compared to the simulated *S*(*q*, ω) as obtained from the MD simulations. The MD simulations were carried out with the gpumd package.^43,44^ The atomic interactions were modeled using a machine-learned potential of the neuroevolution potential (NEP) form, which was constructed in ref (45). This NEP model accurately reproduces energies, forces, and virials from density functional theory calculations based on the vdW-DF-cx functional.^46,47^ A time step of 2 fs was used in the MD simulations. The lattice parameters were determined through *NPT* simulations, after which *S*(*q*, ω) was obtained from *NVE* simulations. In order to sample many different ***q***-points and mimic the experimental results, the *S*(*q*, ω)s were averaged over about 20 different system sizes, ranging from 5000 to 4 50 000 atoms. The coherent part of the dynamical structure factors *S*(***q***, ω) was obtained from the Fourier transform of the intermediate scattering function, *F*(***q***, *t*), which was calculated from the MD trajectories using the dynasor package^48^ as
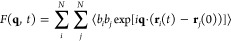
Here, ⟨···⟩ indicates an ensemble average, *N* is the number of atoms, ***r***_*i*_(*t*) is the position of atom *i* at time *t*, and *b*_*i*_ is the bound coherent neutron scattering length of atom *i*. The latter is related to the coherent neutron cross section through σ_coh_ = 4π*b*_*i*_^2^. Since the average incoherent scattering cross section of the nuclei present in CsPbI_3_ is more than a factor 20 smaller than the coherent part, the incoherent dynamical structure *S*(*q*, ω)_inc_ was neglected in the calculation. We employed spherical *q*-point sampling in the range of 0 Å^–1^ to 5 Å^–1^, but we note that the accuracy of *S*(*q*, ω) at *q* ≲ 0.2 Å^–1^ is limited by the finite system size in the MD simulations. For each system size, five independent MD simulations were carried out, each spanning 1 ns. Additionally we also compute the current correlations, *C*(***q***, ω), for both the α- and δ-phase. The current correlation is based on the velocity autocorrelation function and consists of a transverse and a longitudinal part. The longitudinal part is directly related to the dynamical structure factor via
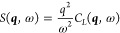
1The current correlation functions are computed with about 2 00 000 atoms along the high-symmetry ***q***-point path of the respective phase and from 7.5 ns long MD simulations.

## Supporting Information Available

Additional experimental details; supplementary INS and MD simulation data (PDF)
