# Supplementary material for: Unraveling the Nature of Vibrational Dynamics in CsPbI3 by Inelastic Neutron Scattering and Molecular Dynamics Simulations
Source: J Phys Chem Lett. 2025 May 8;16(19):4812–8. doi: 10.1021/acs.jpclett.5c00778 (PMC12086850; doi:10.1021/acs.jpclett.5c00778)
Supplement: Supplementary file 1 — jz5c00778_si_001.pdf [file jz5c00778_si_001.pdf]

Supporting Information:
Unraveling the nature of vibrational dynamics in $CsPbI_3$ by inelastic neutron scattering and molecular dynamics simulations

Rasmus Lavén,[1] Erik Fransson,[2] Paul Erhart,[2] Fanni Juranyi,[3] Garrett E. Granroth,[4] and Maths Karlsson[1]

[1]*Department of Chemistry and Chemical Engineering, Chalmers University of Technology, SE-412 96 Göteborg, Sweden.*

[2]*Department of Physics, Chalmers University of Technology, SE-412 96 Göteborg, Sweden.*

[3]*Laboratory for Neutron Scattering and Imaging, PSI Center for Neutron and Muon Sciences, Forschungsstrasse 111, 5232 Villigen, PSI, Switzerland*

[4]*Neutron Scattering Division, Neutron Sciences Directorate, Oak Ridge National Laboratory, Oak Ridge, Tennessee 37831, USA*

CONTENTS

S1. Experimental details S3
A. Samples S3
B. Inelastic neutron scattering S3

S2. Supplementary INS data S4
A. Energy-integrated INS data S4
B. Generalized density of states S4
C. INS data with larger dynamical range S5
D. Temperature dependence of the phonons in $\delta-CsPbI_3$ S6
E. Momentum transfer dependence of the INS signal S7
F. Fitting of $S(q,\omega)$ for cubic perovskites phases S8
G. INS results on $CsPbBr_3$ S9

S3. Supplementary simulation data S10
A. Cross-terms of simulated $S(q,\omega)$ S10
B. Analysis of relaxational dynamics in simulated $S(q,\omega)$ and Comparison of single crystal and powder simulations S11

References S14

S1. EXPERIMENTAL DETAILS

A. Samples

The samples, 12 g of $CsPbI_3$ powder (purity >99%) and 3 g of $CsPbBr_3$ powder (purity >99%), were purchased from Xi'an Polymer Light Technologies and used as received. The phase purity and crystallinity of the samples were confirmed at ambient temperature by powder X-ray diffraction. In addition, for $CsPbI_3$, the phase transition behaviour upon heating the sample from 293 to 650 K was checked using differential scanning calorimetry, which revealed a single endothermic peak at 601 K corresponding to the δ-α phase transition in accordance with the literature [S1, S2].

B. Inelastic neutron scattering

For the experiment on FOCUS we used 9 g of $CsPbI_3$ powder, loaded into a cylindrical 12.5 mm diameter Al sample holder. The measurements were performed using incident neutron wavelengths of 4 Å and 2 Å, respectively, which were obtained from the (002) and (004) Bragg reflections of the pyrolytic graphite monochromator. Using 4 Å incident wavelength neutrons provided an energy resolution at FWHM and wavevector transfer (q) range of approximately 0.2 meV and 0.5 to 2.7 Å^{-1} at the elastic line. For 2 Å incident wavelength neutrons, the corresponding values were about 1 meV and 0.8 to 5.7 Å^{-1}, respectively. Measurements were taken at 50, 300, 400, 500, 550, and 600 K, in that order, with a measurement time of about 4 h per spectrum.

For the experiment on ARCS, we used 4 g of $CsPbI_3$ powder sample (from a different batch), loaded into a cylindrical 6.35 mm diameter Al sample holder. The measurements were performed using incident neutron energies of 15, 30, and 50 meV, which provided the corresponding energy resolutions at full width at half maximum (FWHM) of 0.45, 0.9, and 1.5 meV at the elastic line. Measurements were taken at 10, 300, 400, 495, 539, 564, 580, 595 and 610 K (on heating), and at 500 and 400 K (on cooling), with a measurement time of about 2 h per spectrum. Data reduction was done within the Mantid software [S3] and included normalization to a vanadium standard [S4] and subtraction of an empty sample cell measurement.

Both experiments, on FOCUS and ARCS, used a closed-cycle refrigerator with a high-temperature stage for temperature control. A recent publication from ARCS used the identical temperature controller as we did and we found a temperature-dependent offset between the temperature set point and actual temperature in the experiment [S5]. Using the expression for this offset, as reported in ref. [S5], we re-calibrated our temperature. It is likely that the temperature in the ARCS experiment was about 30 K lower at the highest measured temperature of 640 K, which is in agreement with the observed phase transition behaviour in our experiment. Similarly, for FOCUS, the temperature was re-calibrated by determining the cell parameter of the Al sample cell. Then, the temperature was calibrated to the predicted thermal expansion of Aluminium according to ref. [S6]. Figure S1 shows the re-calibrated temperature versus the read temperature for the FOCUS and ARCS experiment, respectively.

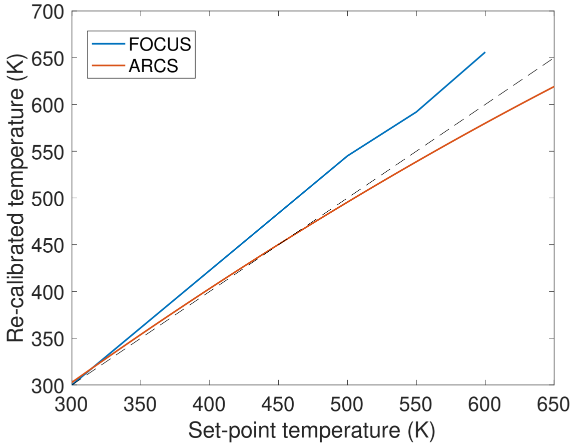

FIG. S1. Re-calibrated temperature versus the set-point temperature for the FOCUS and ARCS experiments. For comparison, the black dashed line has a slope 1.

S2. SUPPLEMENTARY INS DATA

A. Energy-integrated INS data

The studied crystal phases of $CsPbI_3$ during the INS measurements were further confirmed by the elastic Bragg scattering. Figure S2 shows the energy-integrated scattering in the low-q region, showing the Bragg scattering of $CsPbI_3$ for the measurements on ARCS and FOCUS. Upon heating, we observed the δ-α phase transition at around 600 K. Upon cooling ($\approx$ 4 K/min) from the α-phase, we observed the transition directly back to the $\delta-$phase around 540 K (Fig. S3). The phase transition behaviour as observed here is similar to the one reported by Liu *et al.* [S7], but different from other experiments [S8]. These differences may be related to different sample environments, experimental conditions, and sample to sample variations.

B. Generalized density of states

Figure S4 shows the generalized density of states (GDOS) of δ-$CsPbI_3$ at 300 and 550 K and α-$CsPbI_3$ at 600 K. The GDOS was derived from the INS data according to the incoherent approximation [S9, S10], *i.e.*

$$G(\omega) \propto \int \frac{\omega}{q^2} S(q,\omega)(1-\mathrm{e}^{-\hbar\omega/k_\mathrm{B}T})\mathrm{d}q,$$

where k_B is Boltzmann's constant, $\hbar$ is the reduced Planck constant, and T is the temperature.

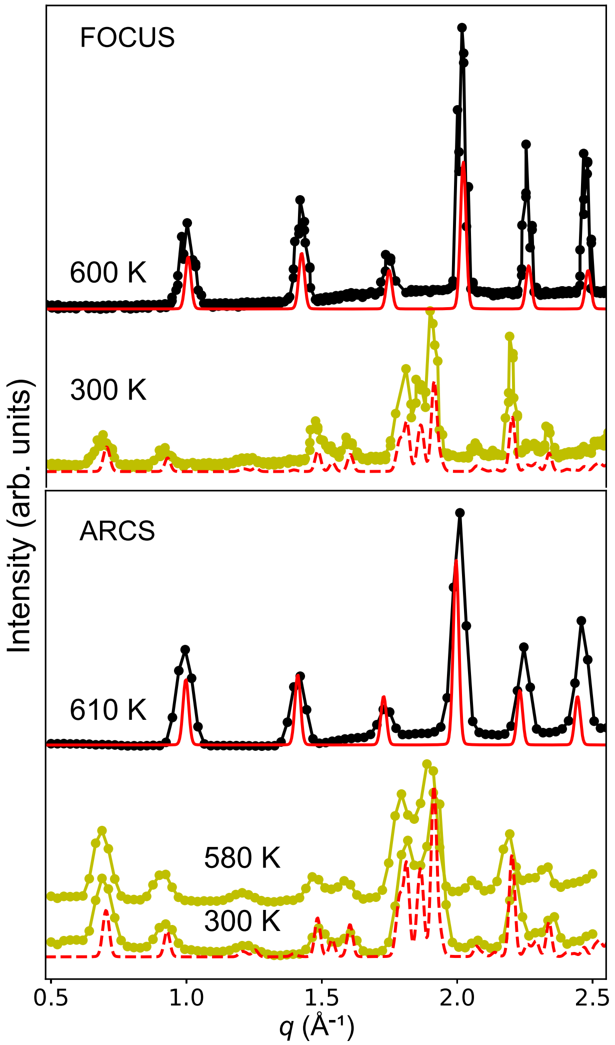

FIG. S2. Energy integrated scattering in the low-q region, as measured on FOCUS (upper panel) using an incident neutron wavelength of 4 Å, and on ARCS (lower panel) using an incident neutron energy of 15 meV, showing the δ-α phase transition. The data is compared to the simulated Bragg scattering of neutrons for the two different phases (dashed red lines for the δ-phase and full red lines for the α-phase). The increase in diffuse scattering for $q > 1.5$ Å^{-1} in the α-phase originates from inelastic scattering. The data sets have been separated vertically. The data were measured at ARCS using an incident neutron energy of 15 meV.

C. INS data with larger dynamical range

The INS data measured on ARCS with 15 meV incident neutron energy is shown in Fig. S5 for $\delta-CsPbI_3$ at 540 K and $\alpha-CsPbI_3$ at 610 K

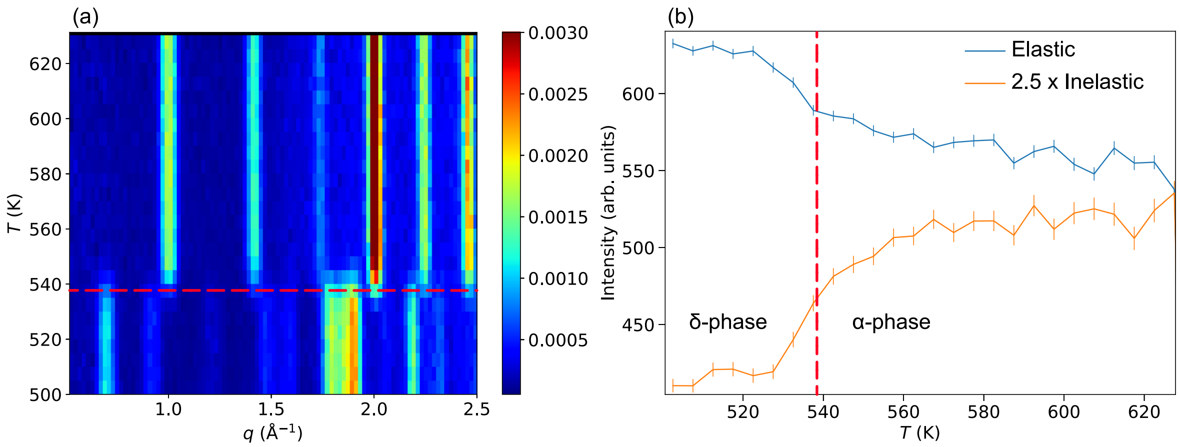

FIG. S3. (a) Energy integrated ($[-10, 10]$ meV) scattering in the low-q region, as measured on ARCS (left panel) using an incident neutron energy of 15 meV during cooling (4 K/min) from 620 K to 500 K. (b) Elastic scattering intensity (integrated over the interval $[-0.5, 0.5]$ meV) and inelastic scattering intensity (integrated over the interval $[0.7, 10]$ meV) integrated over the $q-$range $1-5$ Å^{-1}. The INS intensity is multiplied by a factor of 2.5 for increased visibility. The red dashed lines indicate approximately the transition temperature to the $\delta-$phase.

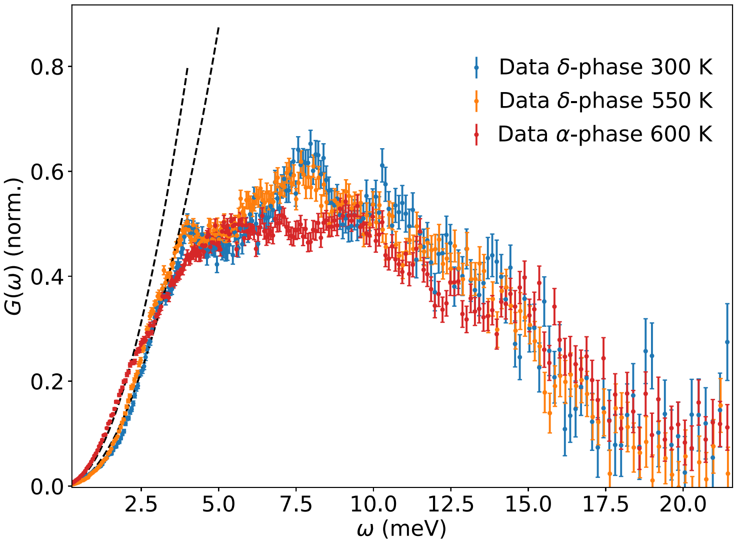

FIG. S4. GDOS of δ-$CsPbI_3$ (300 K and 550 K) and α-$CsPbI_3$ (600 K), as measured on FOCUS with 4 Å incident wavelength neutrons. The GDOS have been normalized to the same number of vibrational modes in the energy range between 0 and 20 meV. The black dashed lines indicate a ω^2 Debye law from acoustic phonons.

D. Temperature dependence of the phonons in $\delta-CsPbI_3$

Figure S6 shows $S(\omega)$ (summed over q), and the corresponding $\chi''(\omega) = S(q, \omega)/(1 + n(\omega))$, for $\delta-CsPbI_3$ for different temperatures from 10 K up to close to the phase transition to the $\alpha-$phase. As can be seen, only a weak temperature dependence is observed. In Fig. S6 (b) the phonon population is accounted for, and the decreasing intensity with increasing

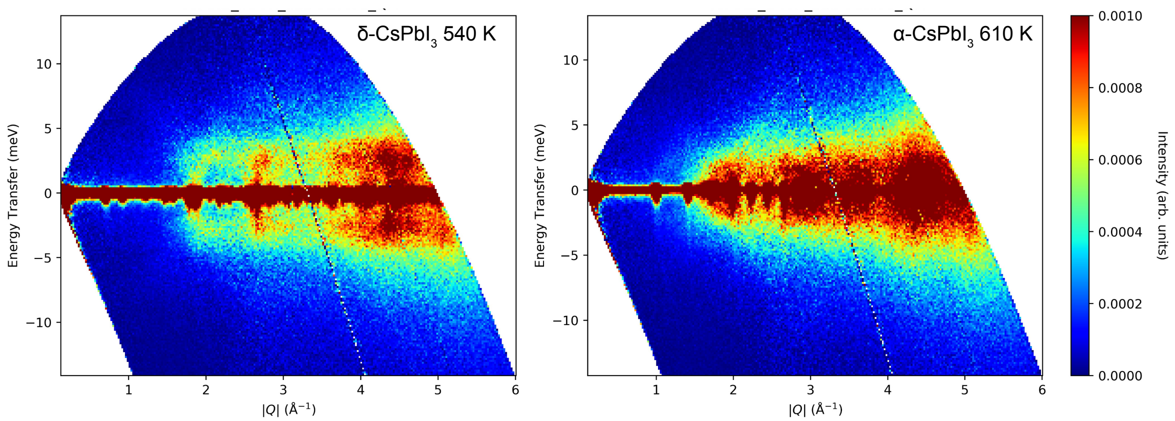

FIG. S5. $S(q, \omega)$ of δ-$CsPbI_3$ (left panel) and α-$CsPbI_3$ (right panel), as measured on ARCS using an incident neutron energy of 15 meV.

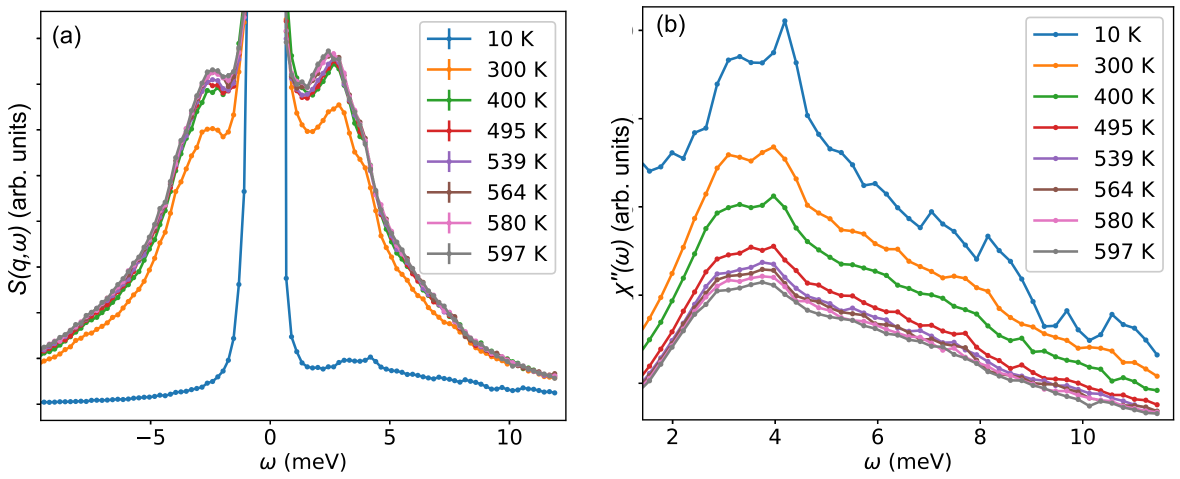

FIG. S6. $S(\omega)$ of δ-$CsPbI_3$ as a function of temperature, and in (b) the corresponding $\chi(\omega)$ accounting for the different phonon population for different temperatures. The data was integrated over q between $1.5 - 5$ Å^{-1}.The data was measured on ARCS using an incident neutron energy of 15 meV.

temperature is due to the increasing Debye-Waller factor.

E. Momentum transfer dependence of the INS signal

Figure S7 shows the $S(q)/q^2$ integrated over the energy interval $[1, 3]$ meV for δ-$CsPbI_3$ and α-$CsPbI_3$. The intensity is divided by q^2 to remove the trivial q^2 increase of phonon scattering. At 540 K, in the δ-phase, $S(q)/q^2$ exhibits a decreasing behaviour with q superimposed with an oscillatory behaviour with local maxima at around 1.9, 2.6, 3.9, and 4.4 Å^{-1}. Such a behaviour is typical for coherent scatterers where the q-behaviour is determined from both an atomic correlation phase factor ($\exp(\mathbf{iq} \cdot \mathbf{r}_j)$) and also the scalar product of $\mathbf{q}$ and the phonon eigenvector [S11]. We note that the overall behaviour of $S(q)/q^2$ is rather similar for both phases, however, there are some important differences. In α-$CsPbI_3$ at 610

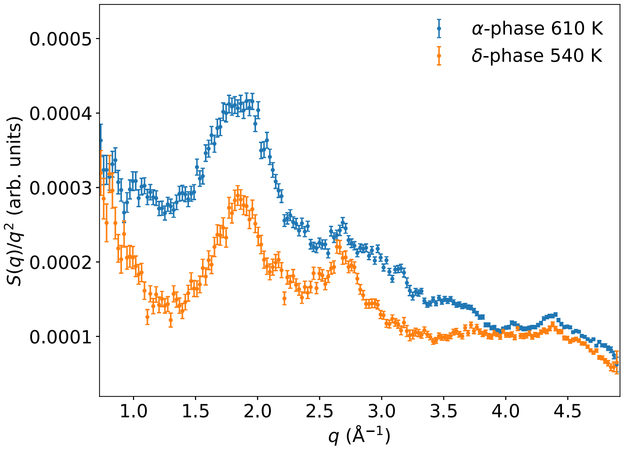

FIG. S7. $S(q)/q^2$ integrated over the energy interval from 1 to 3 meV for δ-CsPbI$_3$ and α-CsPbI$_3$. The data were measured at ARCS using an incident neutron energy of 15 meV.

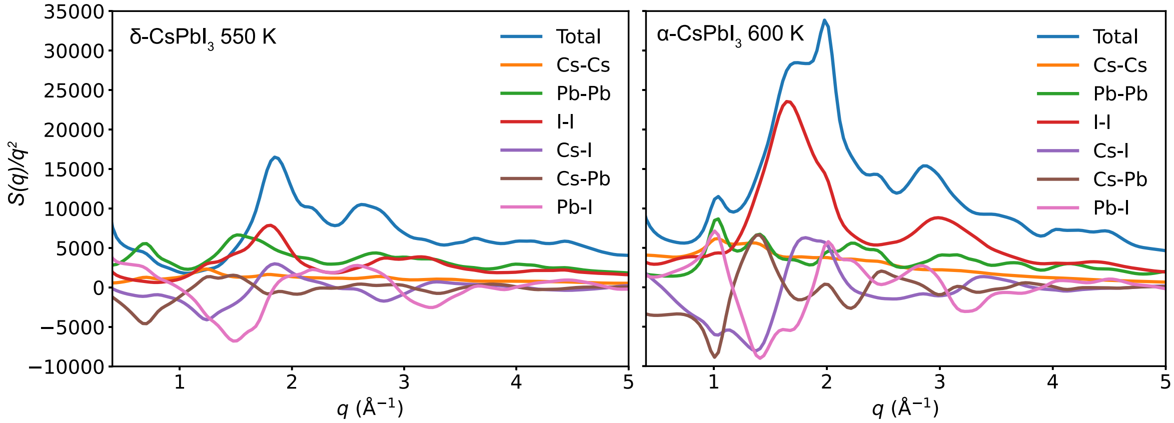

FIG. S8. $S(q)/q^2$ integrated over the energy interval from 0.1 to 3 meV for δ-CsPbI$_3$ and α-CsPbI$_3$, as obtained from the MD simulations. The partial contributions are also shown.

K, this oscillatory behaviour persists, although with slightly broader oscillatory features. Figure S8 shows the corresponding data as obtained from the MD simulations. Shown is also the partial contributions from different atomic correlations. One may note that the Cs-Cs correlations are basically incoherent above ≥ 1.6 Å^{-1} in the $\alpha-$phase. This is more clearly shown in Fig. S9 which shows the partial contributions to $S(q)/q^2$ in the $\delta-$ and $\alpha-$phases.

F. Fitting of $S(q,\omega)$ for cubic perovskites phases

Fitting of the spectra for $\alpha-$CsPbI$_3$ at $q = 2.35$ Å^{-1} and $q = 2.55$ Å^{-1} are shown in Figure S10. As can be seen, no overdamped dynamics can be observed at these q-points. An illustration of the parts of the $q-$ space probed with the powder INS for $q = 1.55$ Å^{-1} and $q = 1.85$ Å^{-1} is shown in Fig. S12. The illustration is a plot of the $L = 0$ plane, and the Brillouin zones are marked as solid black lines.

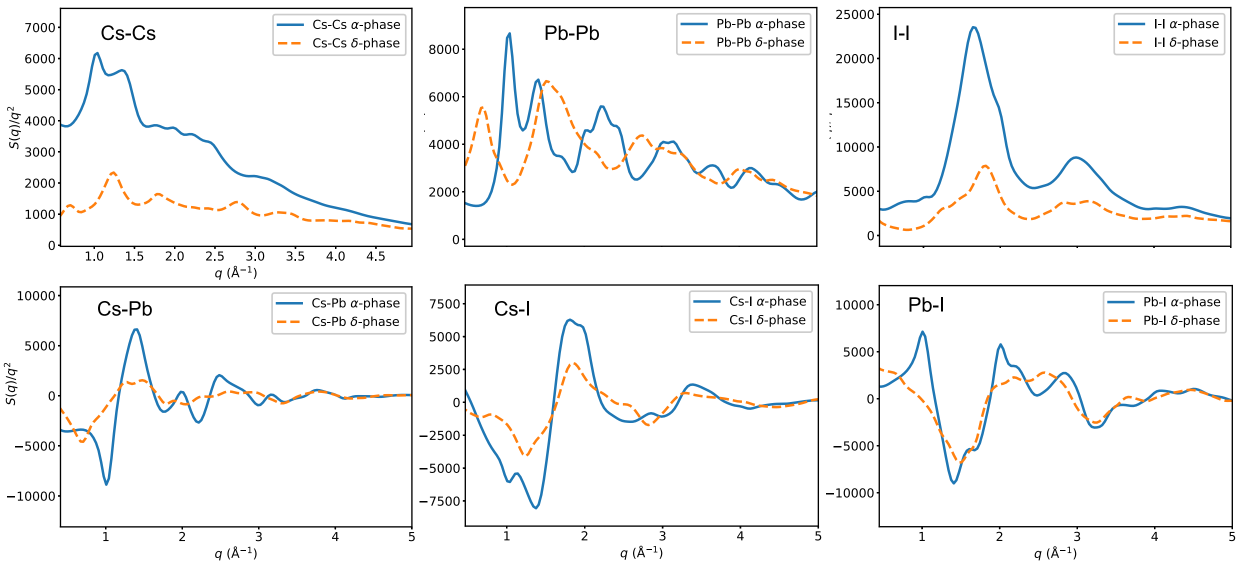

FIG. S9. MD simulated $S(q)/q^2$, integrated over the energy interval from 0.1 to 3 meV, for the different atomic contributions for the $\delta-$CsPbI$_3$ at 550 K and $\alpha-$CsPbI$_3$ at 600 K.

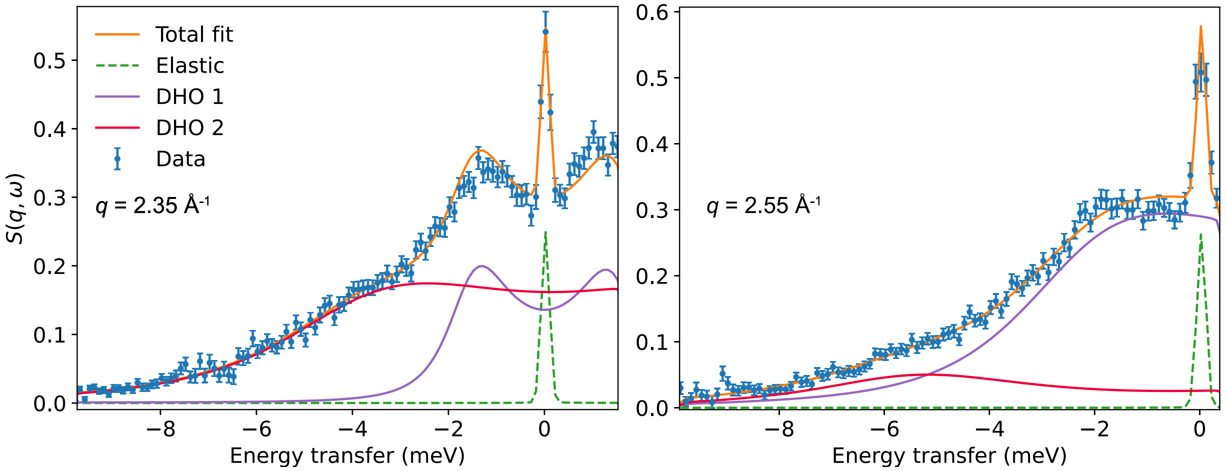

FIG. S10. Fitting of the INS spectra of α-CsPbI$_3$ at 600 K. The data were measured on FOCUS with an incident neutron wavelength of 4 Å.

G. INS results on CsPbBr$_3$

The $S(q, \omega)$ of CsPbBr$_3$ measured at $q = 1.69$ Å^{-1}, roughly corresponding to the modulus of M, as measured on FOCUS with an incident neutron wavelength of 4 Å is shown in Fig. S13. Quasielastic scattering is observed in the tetragonal $\beta-$ and cubic $\alpha-$phases, whereas in the orthorhombic $\gamma-$phase at 300 K, no quasielastic scattering is observed. The spectra can be, similarly to the spectra of CsPbI$_3$, as described in the main paper, be fitted to an elastic component, a quasielastic Lorentzian component, and one damped harmonic oscillator component. The FWHM of the Lorentzian component increases with increases with increasing temperature, and evolves from 0.32 meV at 400 K to 0.57 meV at the

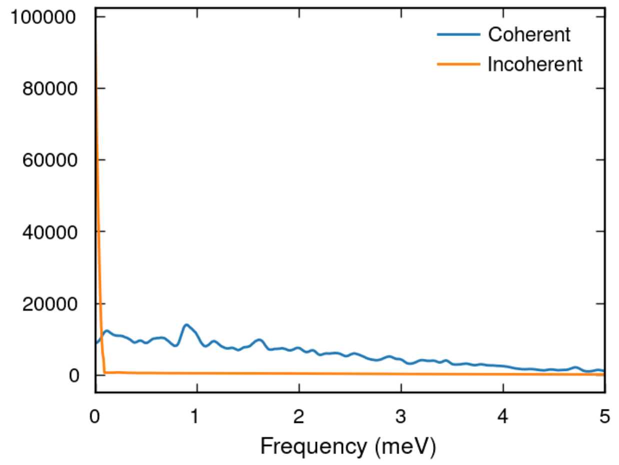

FIG. S11. MD simulated $S(q,\omega)$ at $q = 1.7$ Å^{-1} with both coherent and incoherent contribution. The data is convoluted with a Gaussian of FWHM 0.1 meV. Note that the incoherent mainly contributes to the elastic scattering, whereas the inelastic contribution is negligible.

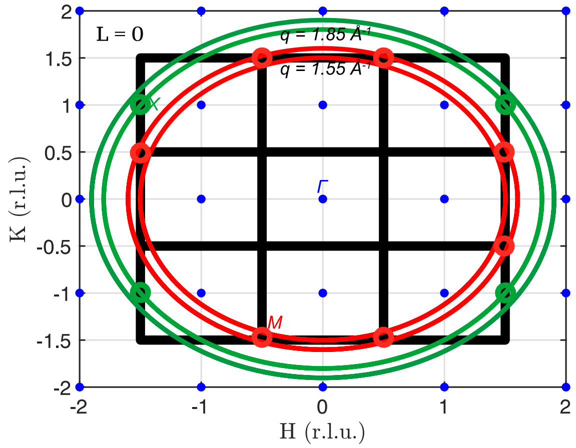

FIG. S12. Illustration of the parts of $q-$space probed with the powder INS for $q = 1.55$ Å^{-1} and $q = 1.85$ Å^{-1}, which is marked as the volume between the red and green lines, respectively. The illustration is a plot of the $L = 0$ plane and the Brillouin zones are marked as solid black lines.

highest measured temperature of 520 K, which corresponds to relaxation times (calculated as $2\hbar/\mathrm{FWHM}$) between $4.2 - 2.3$ ps.

S3. SUPPLEMENTARY SIMULATION DATA

A. Cross-terms of simulated $S(q,\omega)$

The cross-correlation terms of the MD simulated partial dynamical structure factor are shown in Figure S14. Notice that the partial contributions can be negative for some energy and wavevector ranges, but the total $S(q,\omega)$ is always positive. The MD simulated $S(q,\omega)$ at the $M-$point of the Brillouin zone is shown in Figure S15.

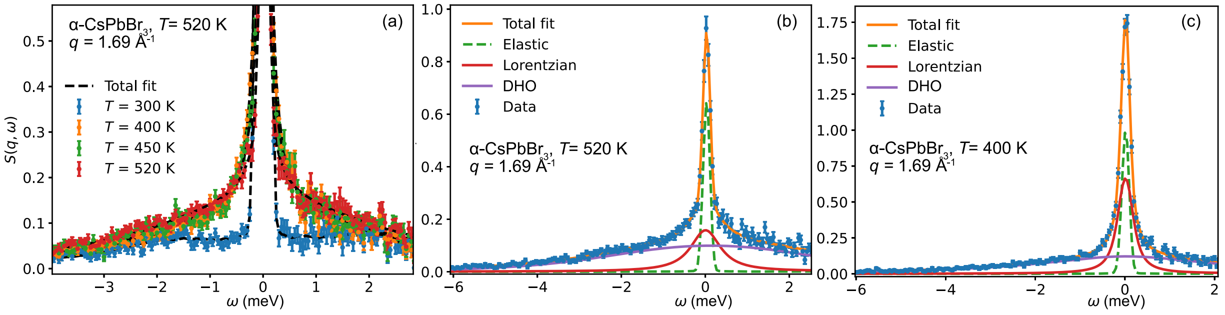

FIG. S13. (a) $S(q,\omega)$ for $CsPbBr_3$ at $q = 1.69$ Å^{-1} for different temperatures. The observed quasielastic scattering is assigned to the M-point overdamped octahedral tilting mode. (b-c) Fitting of the data for 520 K and 400 K. The data was measured on FOCUS using an incident neutron wavelength of 4 Å.

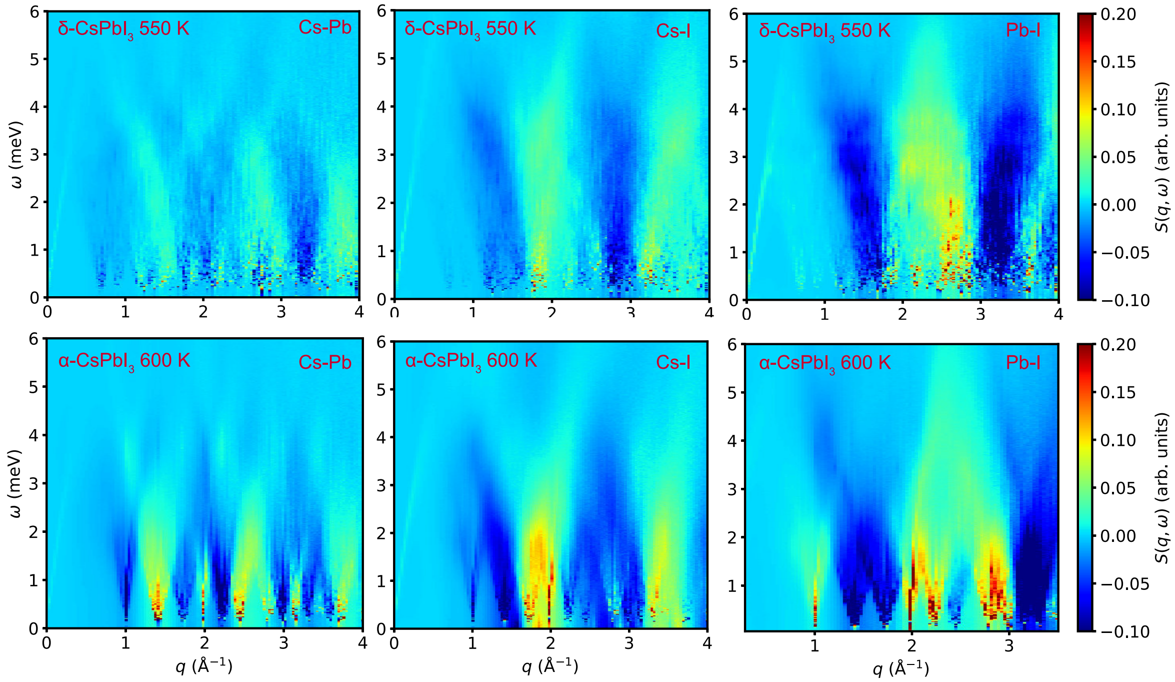

FIG. S14. Cross terms of the MD simulated partial dynamical structure factor for δ-$CsPbI_3$ at 550K (top panels) and α-$CsPbI_3$ at 600K (bottom panels), as calculated from the MD simulations. Note that the partial $S(q,\omega)$s can be negative, but the total $S(q,\omega)$ is always positive.

B. Analysis of relaxational dynamics in simulated $S(q,\omega)$ and Comparison of single crystal and powder simulations

Figure S16 compares the MD simulated partial I-I $S(q,\omega)$ at $M = (3/2, 1/2, 0)$ and the corresponding powder averaged $S(q,\omega)$ at $q = 1.57$ Å^{-1}. Note that the broad peaks centered around ~ 1.2 meV, is only present in the powder averaged $S(q,\omega)$. The observed relaxational dynamics was fitted to a Lorentzian function for with a HWHM of about 0.15 meV at the

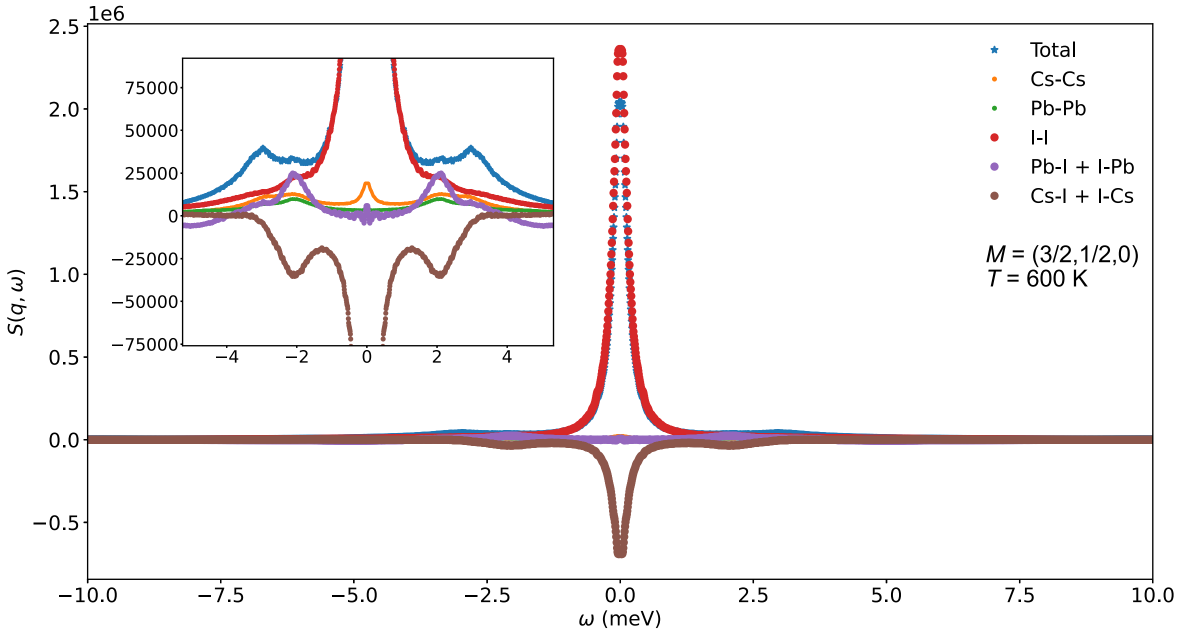

FIG. S15. Partial dynamical structure factor simulated at the M point. The inset shows a close-up of the different atomic contributions. Note that Cs−I shows a strong anti-correlation. The negative frequency side is a mirror image of the positive frequency side, *i.e.* $S(q,-\omega) = S(q,\omega)$.

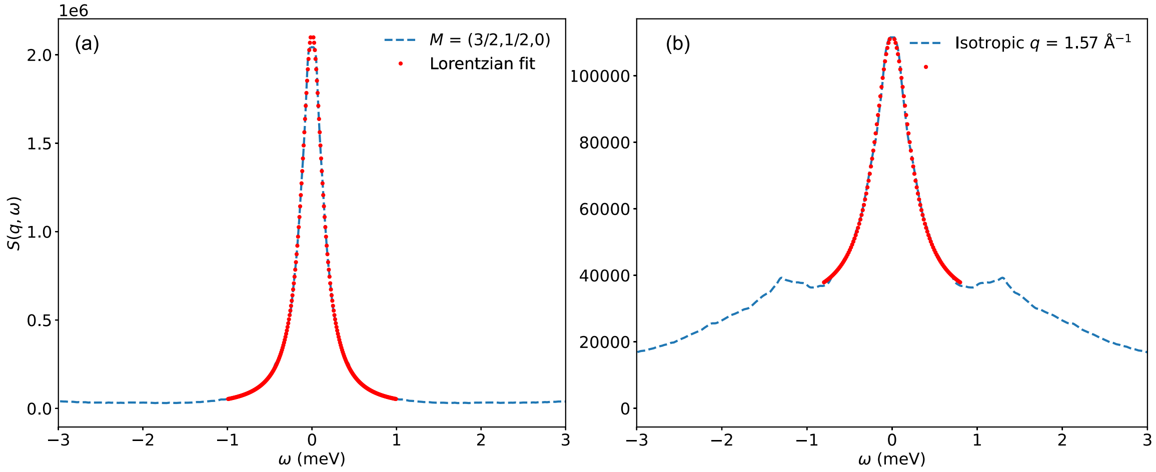

FIG. S16. MD simulated partial I-I $S(q,\omega)$ for (a) at $M = (3/2, 1/2, 0)$ and (b) the corresponding powder averaged $S(q,\omega)$ at $q = 1.57$ Å^{-1}. The observed relaxational dynamics was fitted to a Lorentzian function for with a HWHM of about 0.15 meV for (a) and 0.24 meV for (b). The negative frequency data is a mirror image of the positive frequencies, *i.e.* $S(q,-\omega) = S(q,\omega)$.

M-points and 0.24 meV for the powder averaged $S(q,\omega)$ at $q = 1.57$ Å^{-1}.

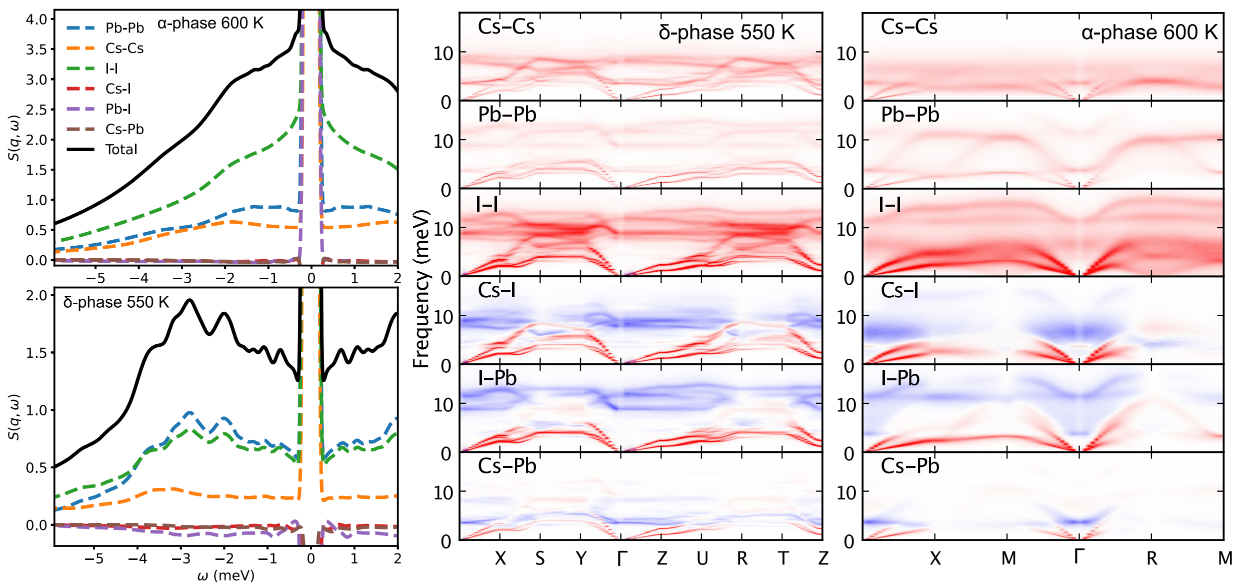

FIG. S17. (Left panel) Partial dynamic structure factors of δ-$CsPbI_3$ at 550 K (bottom) and α-$CsPbI_3$ at 600 K (top) as derived from MD simulations, summed over q from 0.2–3 Å^{-1}. Current correlations for δ-$CsPbI_3$ at 550 K (mid panel) and α-$CsPbI_3$ at 600 K (right panel) as derived from MD simulations, plotted along the high symmetry directions. Note that red and blue colors shows positive and negative values, respectively.

(S1) Dastidar, S.; Hawley, C. J.; Dillon, A. D.; Gutierrez-Perez, A. D.; Spanier, J. E.; Fafarman, A. T. Quantitative Phase-Change Thermodynamics and Metastability of Perovskite-Phase Cesium Lead Iodide. *J. Phys. Chem. Lett.* **2017**, *8*, 1278–1282.

(S2) Wang, B.; Novendra, N.; Navrotsky, A. Energetics, Structures, and Phase Transitions of Cubic and Orthorhombic Cesium Lead Iodide ($CsPbI_3$) Polymorphs. *J. Am. Chem. Soc.* **2019**, *141*, 14501–14504.

(S3) Arnold, O. et al. Mantid - Data analysis and visualization package for neutron scattering and μ SR experiments. *Nucl. Instruments Methods Phys. Res. Sect. A Accel. Spectrometers, Detect. Assoc. Equip.* **2014**, *764*, 156–166.

(S4) Abernathy, D.; Goyette, R.; Granroth, G. ARCS White Beam Vanadium Normalization for SNS Cycle 2021B. Date of Access: 2024-08-25, 2024; doi:10.14461/oncat.data.65a97b8585f57928f1f67178/2281898.

(S5) Miskowiec, A.; Spano, T.; Brubaker, Z. E.; Niedziela, J. L.; Abernathy, D. L.; Hunt, R. D.; Finkeldei, S. Antiferromagnetic ordering and possible lattice response to dynamic uranium valence in U_3O_8. *Phys. Rev. B* **2021**, *103*, 205101.

(S6) Nix, F.; MacNair, D. The thermal expansion of pure metals: copper, gold, aluminum, nickel, and iron. *Phys. Rev.* **1941**, *60*, 597–605.

(S7) Liu, J.; Phillips, A. E.; Keen, D. A.; Dove, M. T. Thermal Disorder and Bond Anharmonicity in Cesium Lead Iodide Studied by Neutron Total Scattering and the Reverse Monte Carlo Method. *J. Phys. Chem. C* **2019**, *123*, 14934–14940.

(S8) Marronnier, A.; Roma, G.; Boyer-Richard, S.; Pedesseau, L.; Jancu, J.-M.; Bonnassieux, Y.; Katan, C.; Stoumpos, C. C.; Kanatzidis, M. G.; Even, J. Anharmonicity and Disorder in the Black Phases of Cesium Lead Iodide Used for Stable Inorganic Perovskite Solar Cells. *ACS Nano* **2018**, *12*, 3477–3486.

(S9) Chazallon, B.; Itoh, H.; Koza, M.; Kuhs, W. F.; Schober, H. Anharmonicity and guest-host coupling in clathrate hydrates. *Phys. Chem. Chem. Phys.* **2002**, *4*, 4809–4816.

(S10) Squires, G. L. *Introduction to the Theory of Thermal Neutron Scattering*; Cambridge University Press, New York, 1978.

(S11) Fabiani, E.; Fontana, A.; Buchenau, U. Neutron scattering study of the vibrations in vitreous silica and germania. *J. Chem. Phys.* **2008**, *128*, 244507.
